# New Supramolecular Hypoxia-Sensitive Complexes Based on Azo-Thiacalixarene

**DOI:** 10.3390/molecules28020466

**Published:** 2023-01-04

**Authors:** Farida Galieva, Mohamed Khalifa, Zaliya Akhmetzyanova, Diana Mironova, Vladimir Burilov, Svetlana Solovieva, Igor Antipin

**Affiliations:** 1 Arbuzov Institute of Organic and Physical Chemistry, FRC Kazan Scientific Center, Russian Academy of Sciences, 420088 Kazan, Russia; 2 Department of Organic and Medical Chemistry, Kazan Federal University, 420008 Kazan, Russia; 3 Chemistry Department, Faculty of Science, Damanhour University, Damanhur 22511, Egypt

**Keywords:** supramolecular chemistry, thiacalixarene, hypoxia sensing, host–guest complex, cationic rhodamine dyes

## Abstract

Hypoxia accompanies many human diseases and is an indicator of tumor aggressiveness. Therefore, measuring hypoxia in vivo is clinically important. Recently, complexes of calix[4]arene were identified as potent hypoxia markers. The subject of this paper is new hypoxia-sensitive host–guest complexes of thiacalix[4]arene. We report a new high-yield synthesis method for thiacalix[4]arene with four anionic carboxyl azo fragments on the upper rim (thiacalixarene **L**) and an assessment of the complexes of thiacalixarene **L** with the most widespread cationic rhodamine dyes (6G, B, and 123) sensitivity to hypoxia. Moreover, 1D and 2D NMR spectroscopy data support the ability of the macrocycles to form complexes with dyes. Rhodamines B and 123 formed host–guest complexes of 1:1 stoichiometry. Complexes of mixed composition were formed with rhodamine 6G. The association constant between thiacalixarene **L** and rhodamine 6G is higher than for other dyes. Thiacalixarene **L**-dye complexes with rhodamine 6G and rhodamine B are stable in the presence of various substances present in a biological environment. The UV-VIS spectrometry and fluorescence showed hypoxia responsiveness of the complexes. Our results demonstrate that thiacalixarene **L** has a stronger binding with dyes compared with the previously reported azo-calix[4]arene carboxylic derivative. Thus, these results suggest higher selective visualization of hypoxia for the complexes with thiacalixarene **L**.

## 1. Introduction

According to the Global Cancer Statistics 2020, there were approximately 19.3 million new cancer cases and almost 10 million cancer deaths worldwide in 2020 [1]. Early diagnosis and treatment of cancer are crucial to reducing global mortality. A common feature of most tumors is low levels of oxygen–hypoxia [2], the severity of which depends on the type of cancer [3]. The need for oxygen exceeds the oxygen supply in the intensively proliferating and growing tumor tissue by increasing the distance in the tissue from the nearest vessel, which prevents oxygen diffusion [4]. Since the discovery of hypoxic regions in tumors in the 1950s, hypoxia has become known as a major hallmark of cancer cells and their microenvironment [5]. Hypoxia facilitates tumor growth, resistance to chemotherapy, and metastasis [6,7]. Areas with oxygen gradients and acute hypoxia appear in the vascular system and blood flow in tumor development, which induces resistance to many anticancer treatments, especially chemotherapy, radiation therapy, and photodynamic therapy [8]. Hypoxic tissues differ from normoxic in physical, chemical, and biological characteristics, such as low pH, increased expression of hypoxia-inducible factor-1α (HIF-1α), etc. [9,10,11].

Moreover, one of these features is an increase in the activity of oxidoreductases: nitroreductase, azoreductase, and quinone oxidoreductase [12]. Most systems proposed in the literature for the detection of hypoxia are associated with nitroaromatic and quinone derivatives [13,14]. The approach using the reduction of azo groups by azoreductase has proven itself as a promising system for visualizing hypoxia [15]. Sodium dithionite (SDT) is commonly used as the azoreductase mimic to evaluate the hypoxia-induced reduction and cleavage of the azo group since SDT can effectively and reductively cleave to the azo group [16,17]. However, most published data concern the creation of sensors utilizing a covalent binding of a dye and an azo derivative. Despite their effectiveness in creating hypoxia-sensitive systems, such sensors have some disadvantages associated with a complex molecular design, multistage, and expensive synthesis. There is also a risk of increasing the toxicity of the covalently bound compound due to the presence of a linker when the dye is introduced, as well as the formation of toxic products during the reduction of the azo group [18,19,20].

**Scheme 1:**
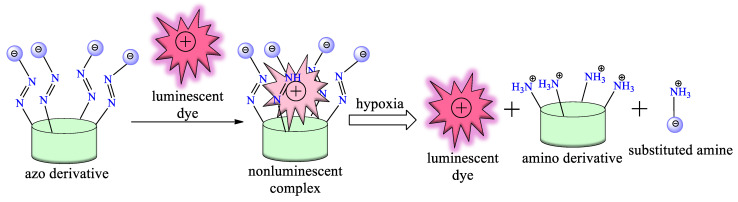
The supramolecular strategy for hypoxia-sensitive systems.

In this regard, the recently appeared supramolecular approach is of particular interest [21]. It consists of developing the systems based on the host–guest interaction between host azo derivatives and guest dyes (Scheme 1). The dye preliminarily interacts with azo derivatives of the macrocycles, forming a host–guest complex. Complex formation leads to quenching of the dye fluorescence. In hypoxic conditions, the azo groups of the macrocycle are reduced to amino groups, which leads to the release of four anionic fragments on the upper rim of the macrocycle and, as a result, the destruction of the host–guest complex and the restoration of dye luminescence. Given that the resulting amines are easily protonated in a wide pH range, they are not able to retain a positively charged dye, which has been shown in a number of works [22,23].

The use of calix[4]arenes as a synthetic platform for the supramolecular approach seems promising due to calixarenes offer several advantages over traditional ligands: the ability to incorporate small organic molecules or a portion of large biomolecules into molecular cavities to form guest–host complexes [24]; the possibility of modification with several receptor groups and fixing their specific spatial arrangement [25,26]; various possibilities in their functionalization both at the upper and lower rims by receptor groups of different nature [27]; the absence of toxicity of both the calixarene platform and a number of derivatives of the latter [28,29,30,31,32]. At the same time, a few examples of the use of macrocycles to create structures capable of detecting and visualizing hypoxia are represented in the literature [33].

**Figure 1:**
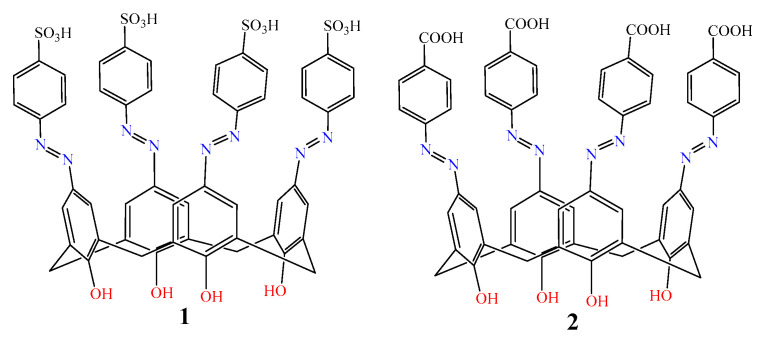
Structures of calix[4]arenes **1, 2** functionalized with azo groups on the upper rims [34].

Recently, our research group proposed supramolecular systems for detecting hypoxia based on calix[4]arenes **1**, **2** functionalized with azo groups on the upper rim (Figure 1) [34]. The present work is devoted to new hypoxia-sensitive systems using the thiacalix[4]arene platform and various rhodamine dyes. The association constant, the stoichiometry of host–guest complexes, and their absorption-emission properties under normoxic and hypoxic conditions are discussed.

## 2. Results

**Scheme 2:**
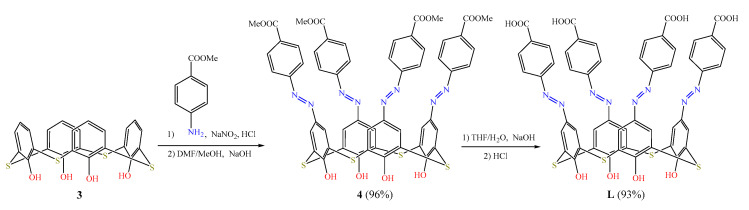
Synthetic pathway for thiacalixarene **L**.

Scheme 2 shows the synthesis of azo-thiacalix[4]arene derivative **L** containing azo benzoic acid fragments. Compound **L** has been synthesized in reference [35]. However, our two-stage synthesis method used in this work provided higher yields of the target product **L**.

**Figure 2:**
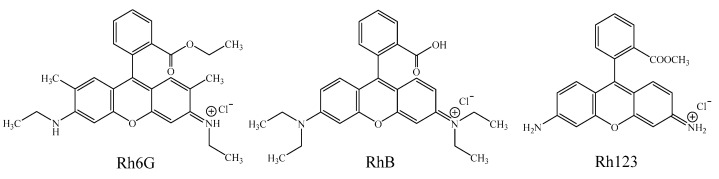
The structures of rhodamine dyes used in binary thiacalix[4]arene-dye systems.

Since the macrocycle **L** contains negatively charged carboxyl groups, the most common cationic dyes, rhodamines 6G, B, and 123, were used for complexation (**Rh6G**, **RhB**, and **Rh123**, Figure 2). The selected dyes are characterized by high luminescence intensity and photostability [36].

**Figure 3:**
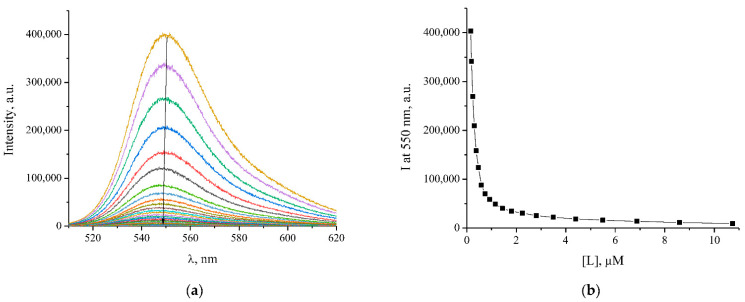
Direct fluorescence titration of **Rh6G** (1.0 μM) with **L** (up to 10.7 μM) (**a**) in PBS buffer (pH 7.4) at 37 °C and the associated titration curve (**b**) at λ_em_ = 550 nm.

The fluorescent response for rhodamine dyes upon the addition of the macrocycle **L** was determined according to fluorescence spectroscopy data. In all macrocycle–dye binary systems, quenching of dye emission was observed with an increase in the macrocycle concentration, which indicates complex formation (Figure 3). In the complex with rhodamine 6G, an increase in the concentration of the macrocycle leads to a slight hypsochromic shift of the dye maximum emission band of about 3 nm. No such effect was observed for **RhB** and **Rh123** (Figure S1).

There are two main types of fluorescence quenching mechanisms in the dye-macrocycle complex–Förster resonance energy transfer (FRET) or photoinduced electron transfer (PET). Previously, our research group showed that for calix[4]arenes **1**, **2** (Figure 1), the PET quenching mechanism prevailed [34].

Thiacalixarene **L** showed a similar trend. The linear dependence of *I*_0_/*I* (*I*_0_–the emission intensity of the free dye, *I*–the emission intensity of the bound dye) on the concentration of the macrocycle (Figure S2) indicates the predominance of only one type of quenching mechanism. The FRET mechanism is excluded due to the absence of overlap in the absorption band of the macrocycle with the emission band of the dye (Figure S3). Therefore, PET is the most likely quenching mechanism. To find the stoichiometry of the dye-macrocycle complexes, Job’s plots were built (Figure S4). The binding stoichiometry for complexes **L** with **RhB** and **Rh123** was 1:1. In the complex of **L** with the **Rh6G** indeterminate composition, an increase in the dye proportion was found. In the complexes of calix[4]arenes **1** and **2** [34], the stoichiometry of complexes with all dyes was found as 1:1. Apparently, the increase in the number of **Rh6G** molecules (coordinated along the upper rim of the macrocycle) was affected by an increase in the size of thiacalix[4]arene cavity. It is obvious that electrostatic interactions are the driving force for the formation of such complexes. However, the preferred interaction of macrocycle **L** with **Rh6G** cannot be explained only from the standpoint of electrostatic interactions. Taking into account the great difference in the logP values of **Rh6G** and **Rh123** (6.5 and 1.5), as well as the difference in logD values (2.1. and 0.5., respectively [37], it can be assumed that the comparatively high hydrophobicity of **Rh6G** favors the interactions with the hydrophobic basket of calixarene. The association constants (*K_ass_*) of azo-thiacalixarene **L** and rhodamine dyes were determined by fitting the data of the fluorescence titration (Table 1).

In the carboxylate calixarene series (**L** and **2**) the higher values of association constants with **Rh6G** were found for thiacalixarene **L**, but in absolute units, the highest association constant was observed in the case of sulfonate derivative **1** with **Rh6G**. This is associated with the electric charge distribution and the structure of the sulfate anion [38,39,40], providing the best electrostatic interactions with cationic dyes compared to carboxylate.

A comparison of absorption spectra for complexes with different dyes showed that **Rh6G** in the presence of **L** had a pronounced hypochromic effect and a 5 nm bathochromic shift of the absorption band of the dye. At the same time, **RhB** in binary **L**–dye complex had only a slight hypochromic shift whereas **Rh123** in the presence of a macrocycle practically had no changes in the absorption spectrum (Figure S5).

**Figure 4:**
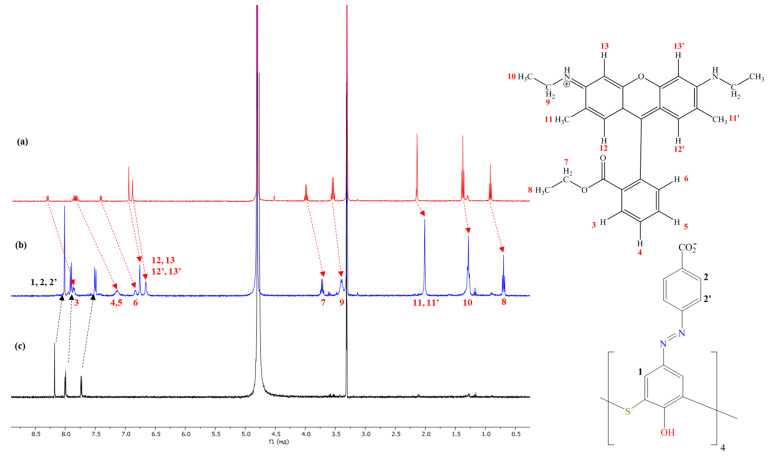
^1^H NMR spectra of 1.25 mM **Rh6G** (**a**), 2.5 mM thiacalixarene **L** (**c**), and their (0.5:1) mixture (**b**) in CD_3_OD-d_4_ at 25 °C.

The ability of macrocycle **L** to form complexes with **Rh6G** was proved by ^1^H NMR spectroscopy. In the presence of **L**, **Rh6G** protons shifts up-field (Figure 4), which is associated with shielding by the macrocycle cavity and indicates the formation of a host–guest complex with partial inclusion of the dye into the macrocyclic core.

The largest shifts were found for the protons of the ester-containing aromatic fragment of the **Rh6G** dye (protons 3, 4, 5, and 6 have Δδ = 0.44, 0.71, and 0.27 ppm, respectively) as well as for protons of the ethoxy group (7 and 8 have Δδ = 0.28 and 0.30 ppm, respectively). The protons of the aminoethyl and the xanthene fragments also undergo an upfield shift (protons 9, 10, 12, 13, and 11 have Δδ = 0.15, 0.20, 0.19, 0.32, and 0.09 ppm, respectively). Thus, it becomes apparent that the dye is immersed in the cavity of the macrocycle, forming a guest-host complex. It should be noted that the up-field shifts of dye proton signals are smaller than those for sulfonate calix[4]arene **1** [34]. However, the thiacalixarene **L** complex demonstrated much larger up-field shifts of dye proton signals compared to the complex of carboxy calix[4]arene **2**-**Rh123** system previously studied by the Go group [33]. Thus, the macrocycle **L** has a stronger binding than calix[4]arene **2** even though they are both bearing negatively charged carboxyl groups. This may be the result of the larger ring size of thiacalix[4]arene, which is 15% larger than that of classical calix[4]arene. Therefore thiacalix[4]arene scaffold is more flexible and easily undergoes conformational changes during the complexation. Due to the high flexibility of the molecular structure thiacalixarenes are superior to “classical” calixarenes for the formation of host–guest complexes [41]. This is consistent with the stability constants of the complexes on the thiacalix[4]arene platform and indicates a stronger interaction of carboxylate thiacalixarene **L** with rhodamine dyes. The interaction of macrocycle **L** with **Rh6G** was also confirmed by the 2D NOESY ^1^H-^1^H spectroscopy (Figure S6). The presence of cross peaks between aromatic protons 1 thiacalixarene **L** (δ = 7.96 ppm) with the **Rh6G** protons 3 (δ = 7.79 ppm), 6 (δ = 6.79 ppm) as well as with of protons the xanthate fragment 12-12’ (δ = 6.72 ppm), 11 (δ = 1.98 ppm), protons of the aminoethyl fragment 10 (δ = 1.27 ppm) unequivocally indicates the formation of the host–guest complex. In addition, cross peaks are observed between protons 2, 2′ of thiacalixarene **L** and protons 3, 6, 11-11’, 13-13’, and 12-12’ (δ = 7.50 with 1.98, 6.60, and 6.72 ppm, respectively). This is consistent with the up-field proton shifts in ^1^H NMR spectra (Figure 4). Thus, it can be concluded that in the complex with thiacalixarene **L**, the **Rh6G** dye is immersed in the cavity mainly by the xanthic part, while in the case of calixarene **1** the same xanthic part remains outside in the complex [34].

The strong complexation in the macrocycle–dye system is a positive feature as it reduces the undesirable slight dissociation of the carrier probe when used in difficult biological conditions. Therefore, all binary macrocycle–dye systems were tested for the presence of a fluorescent response toward various biomolecules and metal ions (Figure S7).

The intensity of dye emission does not change when the third component is added to binary systems with high values of K_a_. It indicates the absence of competitive interaction between the introduced analyte and the dye in the dye–macrocycle system. Thus, systems based on **Rh6G** and **RhB**, which have no competition with bioanalytes and metal ions, are the most promising. For systems with **Rh123**, an increase in emission (up to 100%) is observed due to the displacement of dye from the macrocycle cavity by bioanalytes: lysine, ATP, BSA, and some metals (Na^+^, Ca^2+^).

**Figure 5:**
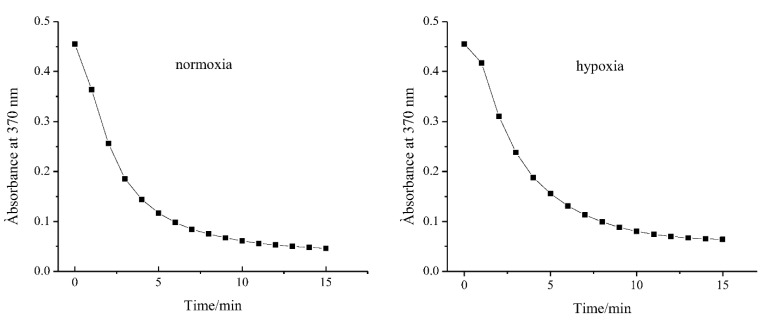
Absorbance at 370 nm of macrocycle **L** (10 μM) as a function of time following the addition of SDT (1.0 mM) under normoxic and hypoxic conditions, PBS buffer, pH 7.4, 37 °C.

UV-visible spectroscopy was used to investigate the hypoxia response of the macrocycle **L**. The thiacalixarene **L** has a broad absorption peak in the range of 300–500 nm, associated with the *n*-π* transitions of the azo groups (Figure S3). The addition of an excess of sodium dithionite (SDT), which acts as a chemical imitator of azoreductase [16,17], to the macrocycle solution causes the disappearance of azo absorption, which indicates the occurrence of a reduction reaction. However, visual discoloration was not observed. Reduction kinetics were quantified by monitoring the absorbance at 370 nm in real time under normoxic (20% O_2_) and hypoxic (<0.1% O_2_) conditions (Figure 5 and Figure S8).

The intensity decay curves were successfully described by the quasi-first-order reaction decay model. Table 2 presents the calculated values of the reduction constants of the azo group. For thiacalix[4]arene **L** in normal conditions, the reduction constant was higher than for calix[4]arenes **1** and **2**. It is noteworthy that hypoxic conditions lead to an increase in the azo group’s reduction rate.

**Figure 6:**
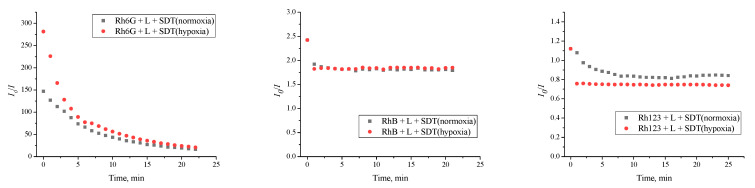
*I*_0_/*I* vs time for rhodamine dye-thiacalixarene **L** systems in the presence of SDT under normoxia and hypoxia. *I*_0_ is the fluorescence intensity of the free dye. C (dye) = 1 μM, C (**L**) = 1 μM, C (SDT) = 100 μM, PBS buffer (pH 7.4).

In passing to binary macrocycle–dye systems, it is anticipated the interaction of the rhodamine dye itself with SDT would make a significant contribution (as shown earlier [34]), leading to the quenching of the luminescence (Figure 6).

For the **RhB**–macrocycle **L** complex, the luminescence was restored to 50% (Figure S9b) of the intensity of the free dye during the first minutes, after which the luminescence intensity of the system remained unchanged for 20 min. At the same time, the results were the same in both normoxia and hypoxia. For the rhodamine-123-macrocycle complex, an increase in luminescence up to 120% of the intensity of the free dye was observed at the 8th minute in normoxic conditions and after a minute in hypoxic (Figure S9c). However, taking into account the weak complexes formation of thiacalixarene **L** with **RhB** and **Rh123**, the increase in the luminescence intensity of the systems was insignificant (Figure 6). In the complex **L** with **Rh6G**, an increase in the luminescence intensity of the system was observed within 20 min. However, the luminescence intensity reached only 5% of the intensity for the free dye in both normoxia and hypoxia (Figure S9a). At the same time, the luminescence quenching of the complex **L**–**Rh6G** is more pronounced before the addition of sodium dithionite in hypoxia. The incomplete restoration of luminescence is apparently due to **Rh6G** interacting with SDT and partially transforming into the leuco form [34]. **Rh6G** luminescence quenching by SDT in the **L**–**Rh6G** system is less pronounced compared with calixarenes **1**,**2**–**Rh6G** complex [34]. Given the fact that even an incomplete restoration of luminescence is an 8-fold (in normoxia) and a 14-fold (in hypoxia) increase in intensity compared with the non-luminescent complex, the thiacalixarene **L**-**Rh6G** complex potentially can be used as a signaling system for hypoxia.

**Figure 7:**
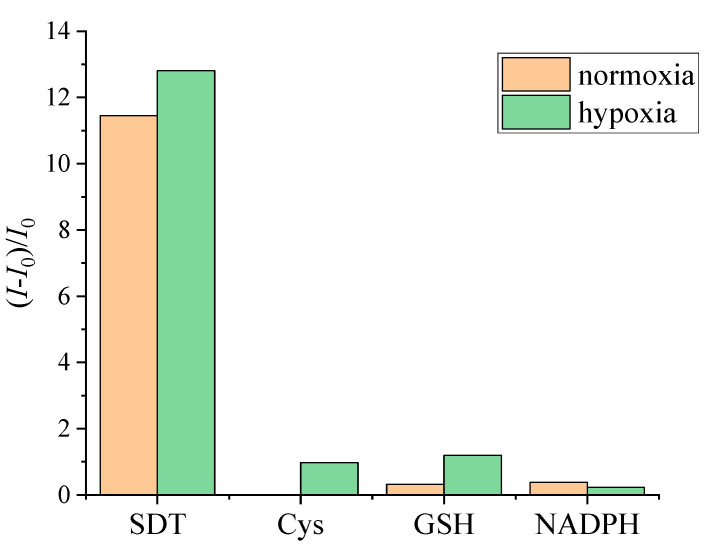
Fluorescent responses of **Rh6G**-**L** (30/10 μM) at 545 nm (λex = 490 nm) to various reductants. The complex was treated with either SDT or various biological reductants (Cys: Cysteine, GSH: glutathione, NADPH: reduced nicotinamide adenine dinucleotide phosphate) under normoxic or hypoxic conditions. Incubation time is 25 min. C (**Rh6G**) = 10 μM, C (**L**) = 30 μM, C (reductants) = 100 μM, PBS buffer (pH 7.4) at 37 °C.

To test the specificity of the response to hypoxia, fluorescence recovery was investigated by replacing SDT with other cellular reductants (cysteine, glutathione, and reduced nicotinamide adenine dinucleotide phosphate) both in hypoxic and normoxic conditions (Figure 7). Obtained emission spectra demonstrate no fluorescent changes in the presence of an excess of selected cellular reductants. The comparison revealed that the binary macrocycle **L**–**Rh6G** system specifically reacts to SDT, which makes it possible to classify this system as a candidate for a hypoxia-sensitive sensor.

## 3. Materials and Methods

### 3.1. Sample Preparation

The phosphate-buffered saline (PBS) solution with pH 7.4 was prepared by dissolving powder (Sigma P-3813) in 1000 mL of Milli-Q water. The stock solutions (100 μM) of macrocycles and dyes were prepared by dissolving the corresponding chemicals in PBS buffer. The fluorescence titrations were performed by successive addition of known amounts of macrocycle to the dye solution in a quartz cuvette. Argon gas was bubbled into the solution for 30 min to create a hypoxic environment.

### 3.2. Apparatus

The NMR spectra were recorded on Bruker Avance 400, 500, and 600 Nanobay (Bruker Corporation, Billerica, MA, USA) with signals from residual protons of CD_3_OD-d_4_ or DMSO-d_6_ solvent as the internal standard. The UV-Vis spectra were recorded in a quartz cell (light path 10 mm) on a Shimadzu UV-2600 UV-Vis spectrophotometer (Shimadzu Corporation, Kyoto, Japan) equipped with a Cary dual-cell Peltier accessory. The fluorescence measurements were recorded in a conventional quartz cell (light path 10 mm) on a Fluorolog FL-221 spectrofluorometer (Horiba, Ltd., Kyoto, Japan) equipped with a single-cell Peltier accessory. MALDI mass spectra were obtained using an UltraFlex III TOF/ TOF spectrometer in the linear mode; *p*-nitroaniline was used as the matrix. Elemental analysis was performed using a PerkinElmer PE 2400 СHNS/О analyzer.

### 3.3. Synthesis

All reagents and solvents were purchased from either Acros (Geel, Belgium) or Sigma-Aldrich (Burlington, VT, USA) and used without further purification. The 5,11,17,23-tetrakis[(4-carboxyphenyl)azo]-25,26,27,28-tetrahydrothiacalix[4]arene **L** [35] was synthesized according to the reported methods. 5,11,17,23-tetrakis-[(4-methoxycarbonyl)-phenylazo]-25,26,27,28-tetrahydroxy-2,8,14,20-tetrathiacalix[4]arene **4**.

NaOH (25 mmol) was added to a solution of 0.248 g (0.5 mmol) of thiacalix[4]arene **3** in 30 mL of a mixture of MeOH and DMF (5:8) with constant stirring in an ice bath. The diazonium salt was obtained by diazotization of aniline benzoate (3 mmol) in 10 mL of a mixture of acetic and hydrochloric acids (1:1), water (5 mL), and NaNO_2_ (3 mmol). The diazonium salt solution was added dropwise. The reaction mixture was stirred for 3 h at 0–5 °C, 10 h at room temperature, and 3 h when heated to 45 °C. The reaction product was precipitated from the reaction mixture by adding a solution of HCl (1:4), washed with water and methanol, and dried at room temperature for 48 h. Yield 96%. ^1^H NMR (600 MHz, DMSO-d_6_, 25 °C) δH ppm: 3.86 (12H, s, –C(O)O-СН_3_), 7.87 (8H, d, *J* = 8.2 Hz, Ar_azo_-H), 8.07 (8H, d, *J* = 8.2 Hz, Ar_azo_-H), 8.17 (8H, s, Ar-H). MALDI TOF (*m/z*): 1168.0 [M + Н]^+^. Found, %: C 55.47; H 4.49; N 10.31. C_56_H_40_N_8_O_12_S_4_·3H_2_O·2(CH_3_)_2_NC(O)H. Calculated, %: C 55.35; H 4.50; N 10.41.

## 4. Conclusions

In summary, we demonstrated the binary system based on azo-thiacalix[4]arene as a potential hypoxia sensor. A systematic study of the thiacalixarene–rhodamine binary system was performed by using UV-visible and fluorescence spectroscopy as well as NMR techniques. It was established that the binding stoichiometry for complexes with rhodamines B and 123 was 1:1. In the complex with rhodamine 6G, mixed complexes of indeterminate composition are formed. The association constants of thiacalixarene- rhodamine 6G complexes were higher than for other dyes. The stability of macrocycle–dye complexes with rhodamine 6G and rhodamine B was verified with the addition of analytes present in a biological environment. It was shown that the azo groups of the macrocycle are selectively reduced by sodium dithionite, which leads to the release of the dye and the restoration of its fluorescence intensity. In comparison with the carboxylate analog based on the classical calix[4]arene, the azo-thiacalix[4]arene has a stronger binding with rhodamine dyes. We expect that these results could pave the way for highly selective visualization of hypoxia in living systems.

## Figures and Tables

**Table 1:** Association constant (*K_ass_*) of thiacalix[4]arene **L** and calix[4]arene **1**,**2** complexes with rhodamine dyes. C(dye) 1 μM, (macrocycle) 0-50 μM, PBS buffer (pH 7.4), 37 °C; ^1^ [34].

System	*K_ass_*, M^−1^
**L**-**Rh6G**	4.7 ± 0.1 × 10^6^
**L**-**RhB**	8.6 ± 0.2 × 10^4^
**L**-**Rh123**	5.2 ± 0.4 × 10^3^
**1**-**Rh6G** ^1^	1.5 ± 0.1 × 10^7^
**1**-**RhB** ^1^	7.0 ± 0.1 × 10^5^
**1**-**Rh123** ^1^	5.0 ± 0.3 × 10^5^
**2**-**Rh6G** ^1^	1.0 ± 0.4 × 10^5^
**2**-**RhB** ^1^	2.6 ± 0.1 × 10^4^
**2**-**Rh123** ^1^	2.3 ± 0.6 × 10^4^

**Table 2:** Reduction kinetics of the macrocycles **L**, **1,** and **2** in the presence of SDT; ^1^ [34].

Macrocycle	*k*, min^−1^ (Normoxia)	*k*, min^−1^ (Hypoxia)
**L**	0.420 (Adj. R^2^ > 0.987)	0.497 (Adj. R^2^ > 0.985)
**1** ^1^	0.350 (Adj. R^2^ > 0.991)	-
**2** ^1^	0.403 (Adj. R^2^ > 0.987)	-

## Data Availability

The data presented in this study are contained within the article or in the Supplementary Materials, or are available upon request from the corresponding authors (Farida Galieva and Svetlana Solovieva).

## Supplementary Materials

The following supporting information can be downloaded at: https://www.mdpi.com/article/10.3390/molecules28020466/s1, Figure S1. Direct fluorescence titration of Rh123 (a) and RhB (b) with **L** (up to 10.7 μM) in PBS buffer (pH 7.4) at 37 °C; Figure S2. Volmer plots for Rh6G, RhB, and Rh123 with thiacalix[4]arene **L**. C (dye) = 1 μM, PBS buffer (pH 7.4) at 37 °C; Figure S3. Normalized emission spectrum of Rho6G and absorption spectrum of thiacalix[4]arene **L** in PBS buffer (pH 7.4) at 37 °C; Figure S4. Job’s plot for solutions rhodamine dyes and thiacalix[4]arene **L**. C [total] = 2 μM, PBS buffer (pH 7.4) at 37 °C; Figure S5. Absorbance spectra of dyes and binary thiacalix[4]arene **L**-dye systems. C [dye] = [calixarene] = 10 μM, PBS buffer (pH 7.4) at 37 °C; Figure S6. A fragment of 2D NOESY ^1^H-^1^H spectra of thiacalix[4]arene **L** with Rh6G, 1.25 mM Rho6G and 2.5 mM of thiacalix[4]arene **L** in CD_3_OD-d_4_ at 25 °C; Figure S7. Fluorescent response of binary dye–thiacalix[4]arene **L** systems upon the addition of various biologically coexisting species. *I*_0_ and *I* are the fluorescence intensity before and after the addition of various substances present in biological media, respectively. In the last right column, *I* is the fluorescence intensity of the free dyes. C [dye] = 1 μM, C [**L**] = 3 μM, C [substance] = 10 μM, PBS buffer (pH 7.4) at 37 °C. Figure S8. Absorbance spectra of **L** (10 μM) before and after reduction by SDT (1.0 mM) under normoxic (20% O_2_) (a) and hypoxic (less than 0.1% O_2_) (b) conditions for 15 min, PBS buffer (pH 7.4) at 37 °C. Figure S9. Fluorescent spectra of dye, the complex before the addition of SDT, and after 20 min of exposure with the addition of SDT for systems with Rh6G (a), RhB (b), and Rh123 (c). C [dye] = 1 μM, C [**L**] = 1 μM, C [SDT] = 100 μM, PBS buffer (pH 7.4) at 37 °C.

## Sample Availability

Samples of the compound **L** are available from the authors.

## References

[1] Sung H., Ferlay J., Siegel R.L., Laversanne M., Soerjomataram I., Jemal A., Bray F. (2021). Global cancer statistics 2020: GLOBOCAN estimates of incidence and mortality worldwide for 36 cancers in 185 countries. CA A Cancer J. Clin..

[2] Lee J.W., Ko J., Ju C., Eltzschig H.K. (2019). Hypoxia signaling in human diseases and therapeutic targets. Exp. Mol. Med..

[3] Bhandari V., Hoey C., Liu L.Y., Lalonde E., Ray J., Livingstone J., Lesurf R., Shiah Y.J., Vujcic T., Huang X.Y. (2019). Molecular landmarks of tumor hypoxia across cancer types. Nat. Genet..

[4] Semenza G.L. (2000). Hypoxia, clonal selection, and the role of HIF-1 in tumor progression. Crit. Rev. Biochem. Mol. Biol..

[5] Thomlinson R.H., Gray L.H. (1955). The histological structure of some human lung cancers and the possible implications for radiotherapy. Br. J. Cancer.

[6] Hayashi Y., Yokota A., Harada H., Huang G. (2019). Hypoxia/pseudohypoxia-mediated activation of hypoxia-inducible factor-1α in cancer. Cancer Sci..

[7] Gilkes D.M., Semenza G.L. (2013). Role of hypoxia-inducible factors in breast cancer metastasis. Future Oncol..

[8] Brown J.M., William W.R. (2004). Exploiting tumour hypoxia in cancer treatment. Nat. Rev. Cancer.

[9] Petrova V., Annicchiarico-Petruzzelli M., Melino G., Amelio I. (2018). The hypoxic tumour microenvironment. Oncogenesis.

[10] Avagliano A., Granato G., Ruocco M.R., Romano V., Belviso I., Carfora A., Montagnani S., Arcucci A. (2018). Metabolic reprogramming of cancer associated fibroblasts: The slavery of stromal fibroblasts. BioMed Res. Int..

[11] Ammirante M., Shalapour S., Kang Y., Jamieson C.A.M., Karin M. (2014). Tissue injury and hypoxia promote malignant progression of prostate cancer by inducing CXCL13 expression in tumor myofibroblasts. Proc. Natl. Acad. Sci. USA.

[12] Zhou H., Qin F., Chen C. (2021). Designing hypoxia-responsive nanotheranostic agents for tumor imaging and therapy. Adv. Healthc. Mater..

[13] Tanabe K., Hirata N., Harada H., Hiraoka M., Nishimoto S.-I. (2008). Emission under hypoxia: One-electron reduction and fluorescence characteristics of an indolequinone-coumarin conjugate. Chembiochem.

[14] Liu Y., Xu Y.F., Qian X.H., Liu J.W., Shen L.Y., Li J.H., Zhang Y.X. (2006). Novel fluorescent markers for hypoxic cells of naphthalimides with two heterocyclic side chains for bioreductive binding. Bioorg. Med. Chem..

[15] Kumari R., Sunil D., Ningthoujam R.S., Kumar N.V.A. (2019). Azodyes as markers for tumor hypoxia imaging and therapy: An up-to-date review. Chem.-Biol. Interact..

[16] Chevalier A., Mercier C., Saurel L., Orenga S., Renard P.-Y., Romieu A. (2013). The first latent green fluorophores for the detection of azoreductase activity in bacterial cultures. Chem. Commun..

[17] Leriche G., Budin G., Darwich Z., Weltin D., Mély Y., Klymchenko A.S., Wagner A. (2012). A FRET-based probe with a chemically deactivatable quencher. Chem. Commun..

[18] Verwilst P., Han J., Lee J., Mun S., Kang H.-G., Kim J.S. (2017). Reconsidering azobenzene as a component of small-molecule hypoxia-mediated cancer drugs: A theranostic case study. Biomaterials.

[19] Yuan P., Zhang H., Qian L., Mao X., Du S., Yu C., Peng B., Yao S.Q. (2017). Intracellular delivery of functional native antibodies under hypoxic conditions by using a biodegradable silica nanoquencher. Angew. Chem. Int. Ed..

[20] Perche F., Biswas S., Wang T., Zhu L., Torchilin V.P. (2014). Hypoxia-targeted siRNA delivery. Angew. Chem. Int. Ed..

[21] Ma X., Zhao Y.L. (2015). Biomedical applications of supramolecular systems based on host-guest interactions. Chem. Rev..

[22] Chevalier A., Piao W., Hanaoka K., Nagano T., Renard P.-Y., Romieu A. (2015). Azobenzene-caged sulforhodamine dyes: A novel class of ‘turn-on’ reactive probes for hypoxic tumor cell imaging. Methods Appl. Fluores.

[23] Piao W., Tsuda S., Tanaka Y., Maeda S., Liu F., Takahashi S., Kushida Y., Komatsu T., Ueno T., Terai T. (2013). Development of Azo-Based Fluorescent Probes to Detect Different Levels of Hypoxia. Angew. Chem. Int. Ed..

[24] Tauran Y., Coleman A.W., Perret F., Kim B. (2015). Cellular and in vivo biological activities of the calix[n]arenes. Curr. Org. Chem..

[25] Nimse S.B., Kim T. (2013). Biological applications of functionalized calixarenes. Chem. Soc. Rev..

[26] Baldini L., Casnati A., Sansone F. (2020). Multivalent and multifunctional calixarenes in bionanotechnology. Eur. J. Org. Chem..

[27] Tian H.W., Liu Y.C., Guo D.S. (2020). Assembling features of calixarene-based amphiphiles and supra-amphiphiles. Mater. Chem. Front..

[28] Mokhtari B., Pourabdollah K. (2013). Applications of nano-baskets in drug development: High solubility and low toxicity. Drug Chem. Toxicol..

[29] Ukhatskaya E.V., Kurkov S.V., Matthews S.E., Loftsson T. (2013). Encapsulation of drug molecules into calix[n]arene nanobaskets. Role of aminocalix[n]arenes in biopharmaceutical field. J. Pharm. Sci..

[30] Coleman A.W., Jebors S., Cecillon S., Perret P., Garin D., Marti-Battle D., Moulin M. (2008). Toxicity and biodistribution of para-sulfonato-calix [4]arene in mice. New J. Chem..

[31] Perret F., Coleman A.W. (2011). Biochemistry of anionic calix[n]arenes. Chem. Commun..

[32] Perret F., Lazar A.N., Coleman A.W. (2006). Biochemistry of the para-sulfonato-calix[n]arenes. Chem. Commun..

[33] Geng W.-C., Jia S., Zheng Z., Li Z., Ding D., Guo D.-S. (2019). A noncovalent fluorescence turn-on strategy for hypoxia imaging. Angew. Chem. Int. Ed..

[34] Mironova D., Burilov B., Galieva F., Khalifa M.A.M., Kleshnina S., Gazalieva A., Nugmanov R., Solovieva S., Antipin I. (2021). Azocalix[4]arene-rhodamine supramolecular hypoxia-sensitive systems: A search for the best calixarene hosts and rhodamine guests. Molecules.

[35] Chakrabarti A., Chawla H.M., Francis T., Pant N., Upreti S. (2006). Synthesis and cation binding properties of new arylazo- and heteroarylazotetrathiacalix[4]arenes. Tetrahedron.

[36] Rajasekar M. (2021). Recent Trends in Rhodamine derivatives as fluorescent probes for biomaterial applications. J. Mol. Struct..

[37] Kennedy A.R., Conway L.K., Kirkhouse J.B.A., McCarney K.M., Puissegur O., Staunton E., Teat S.J., Warren J.E. (2020). Monosulfonated Azo Dyes: A Crystallographic Study of the Molecular Structures of the Free Acid, Anionic and Dianionic Forms. Crystals.

[38] Zana R., Schmidt J., Talmon Y. (2005). Tetrabutylammonium Alkyl Carboxylate Surfactants in Aqueous Solution: Self-Association Behavior, Solution Nanostructure, and Comparison with Tetrabutylammonium Alkyl Sulfate Surfactants. Langmuir.

[39] Reshetnyak E.A., Chernysheva O.S., Nikitina N.A., Loginova L.P., Mchedlov-Petrosyan N.O. (2014). Activity coefficients of alkyl sulfate and alkylsulfonate ions in aqueous and water-salt premicellar solutions. Colloid J..

[40] Burilov V.A., Mironova D.A., Ibragimova R.R., Nugmanov R.I., Solovieva S.E., Antipin I.S. (2017). Detection of sulfate surface-active substances via fluorescent response using new amphiphilic thiacalix[4]arenes bearing cationic headgroups with Eosin Y dye. Colloids Surf. A Physicochem. Eng. Asp..

[41] Solovieva S.E., Burilov V.A., Antipin I.S. (2017). Thiacalix[4]arene’s Lower Rim Derivatives: Synthesis and Supramolecular Properties. Macroheterocycles.

