# Supplementary material for: New Supramolecular Hypoxia-Sensitive Complexes Based on Azo-Thiacalixarene
Source: Molecules. 2023 Jan 4;28(2):466. doi: 10.3390/molecules28020466 (PMC9862174; doi:10.3390/molecules28020466)
Supplement: Supplementary file 1 [file molecules-28-00466-s001.zip › molecules-2101799-supplementary-update.pdf]

molecules

Supplementary information

New supramolecular hypoxia-sensitive complexes based on azo-thiacalixarene

Farida Galieva, Mohamed Khalifa, Zaliya Akhmetzyanova, Diana Mironova, Vladimir Burilov, Svetlana Solovieva and Igor Antipin

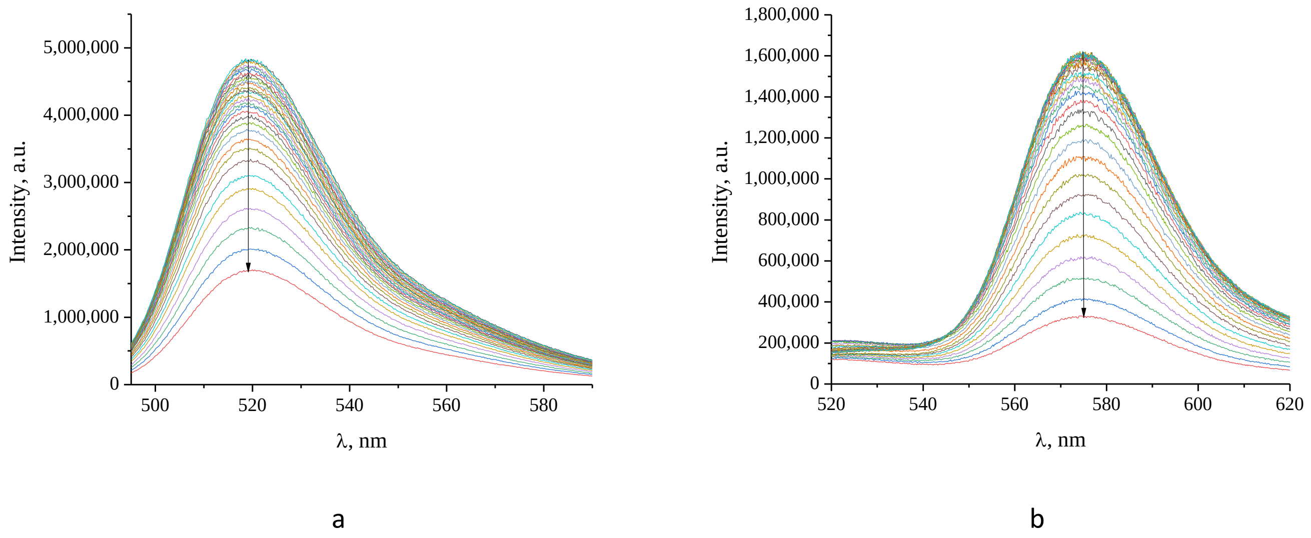

Figure S1. Direct fluorescence titration of **Rh123** (a) and **RhB** (b) with **L** (up to 10.7 μM) in PBS buffer (pH 7.4) at 37°C.

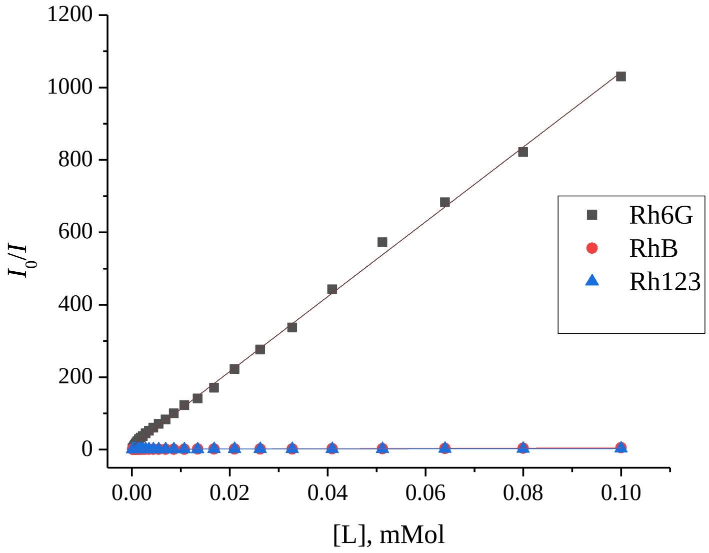

Figure S2. Volmer plots for **Rh6G**, **RhB** and **Rh123** with thiacalix[4]arene **L**. C (dye) = 1 μM, PBS buffer (pH 7.4) at 37°C.

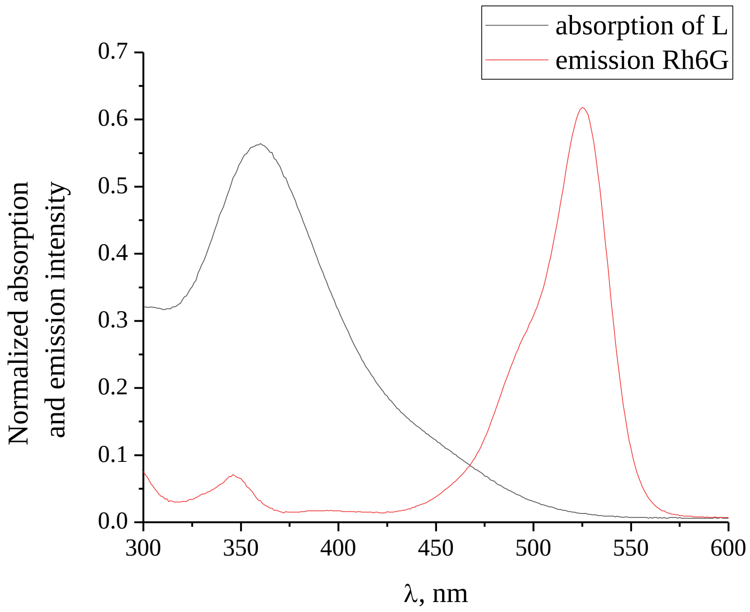

Figure S3. Normalized emission spectrum of **Rh6G** and absorption spectrum of thiacalix[4]arene **L** in PBS buffer (pH 7.4) at 37 °C.

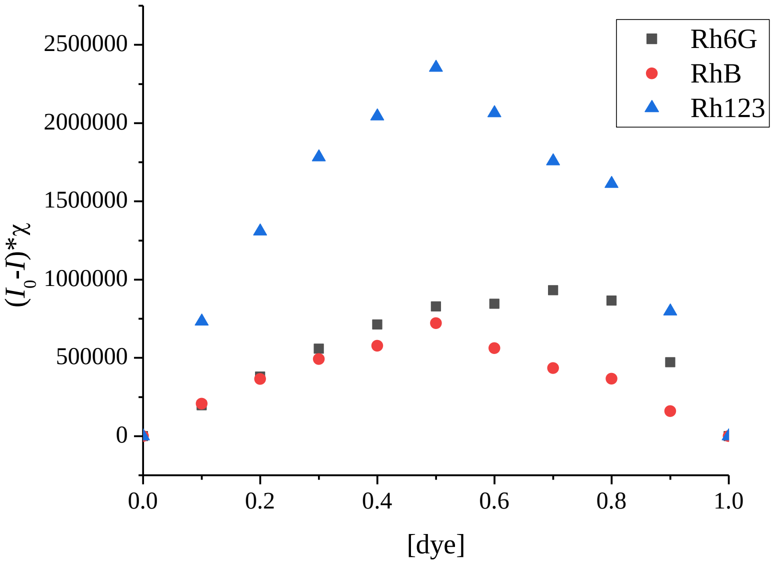

Figure S4. Job's plot for solutions rhodamine dyes and thiacalix[4]arene **L**. C [total] = 2 µM, PBS buffer (pH 7.4) at 37 °C.

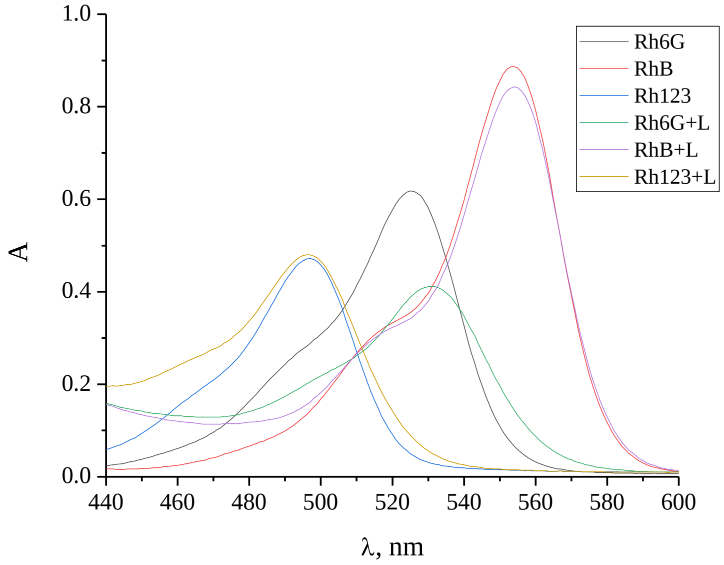

Figure S5. Absorbance spectra of dyes and binary thiacalix[4]arene **L** – dye systems. C [dye] = [calixarene] = 10 μM, PBS buffer (pH 7.4) at 37 °C.

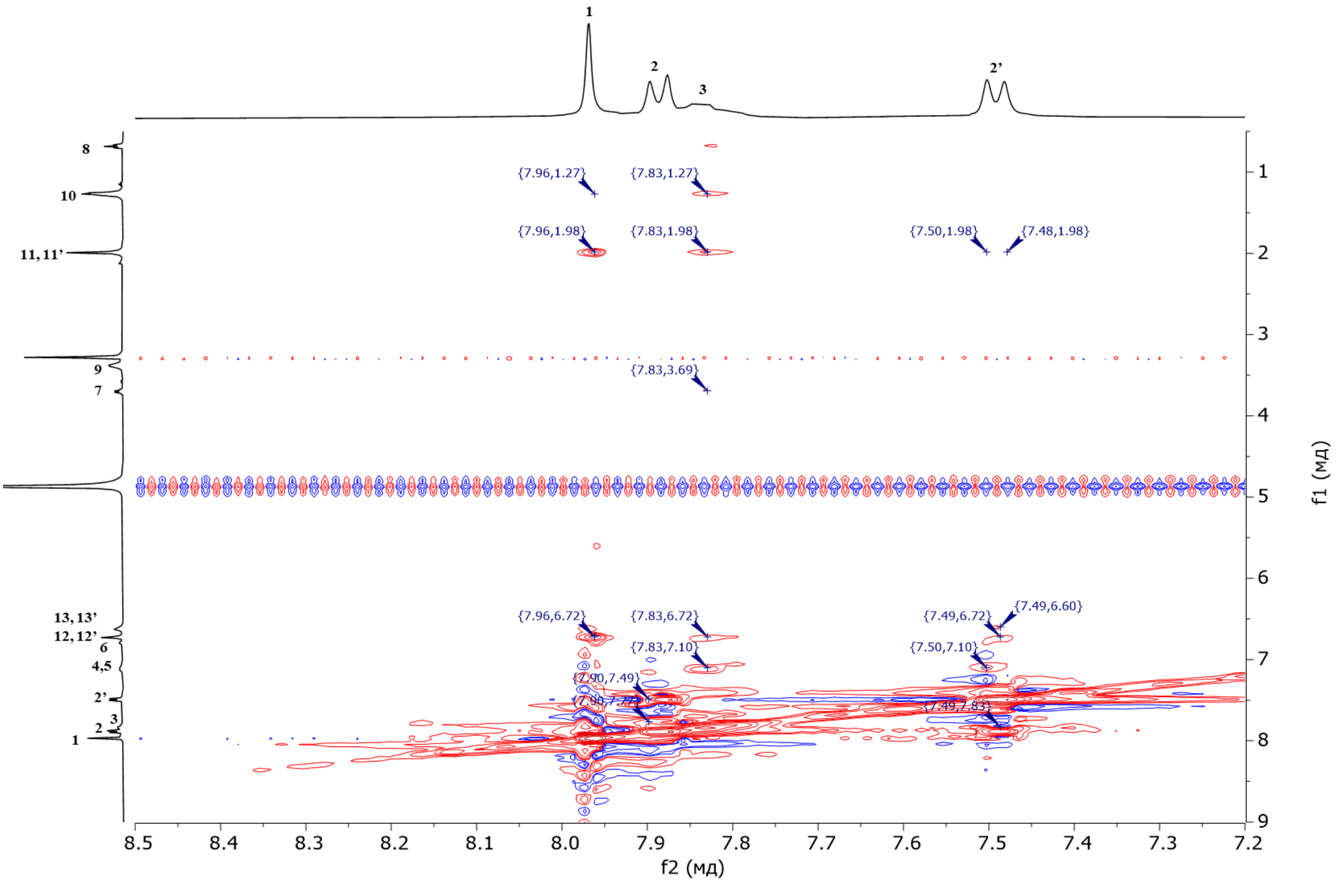

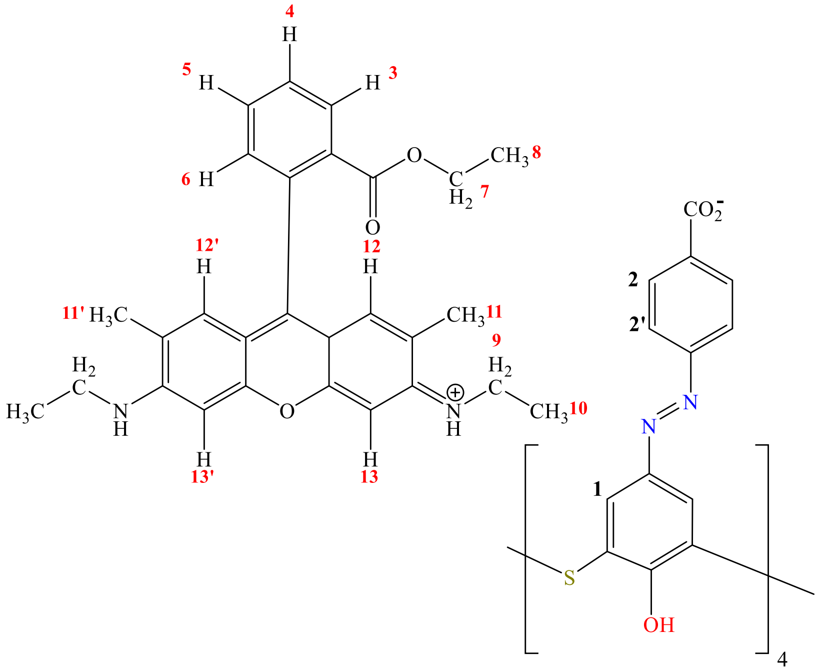

Figure S6. A fragment of 2D NOESY ^1H-^1H spectra of thiacalix[4]arene **L** with **Rh6G**, 1.25 mM Rho6G and 2.5 mM of thiacalix[4]arene **L** in CD_3OD-d_4 at 25 °C.

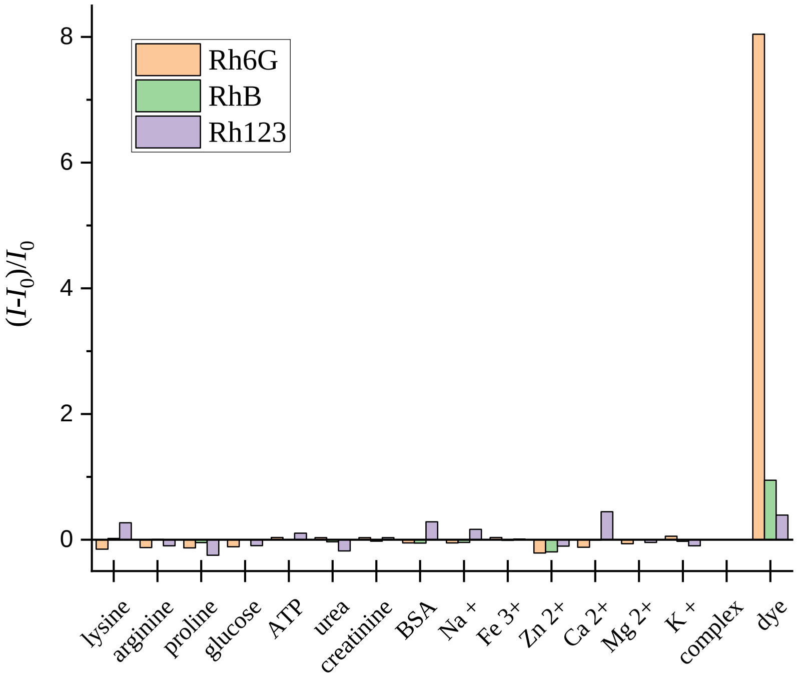

Figure S7. Fluorescent response of binary dye – thiacalix[4]arene **L** systems upon the addition of various biologically coexisting species. I_0 and I are the fluorescence intensity before and after addition of various substances present in biological media, respectively. In the last right column, I is the fluorescence intensity of free dyes. C [dye] = 1 μM, C [**L**] = 3 μM, C [substance] = 10 μM, PBS buffer (pH 7.4) at 37 °C.

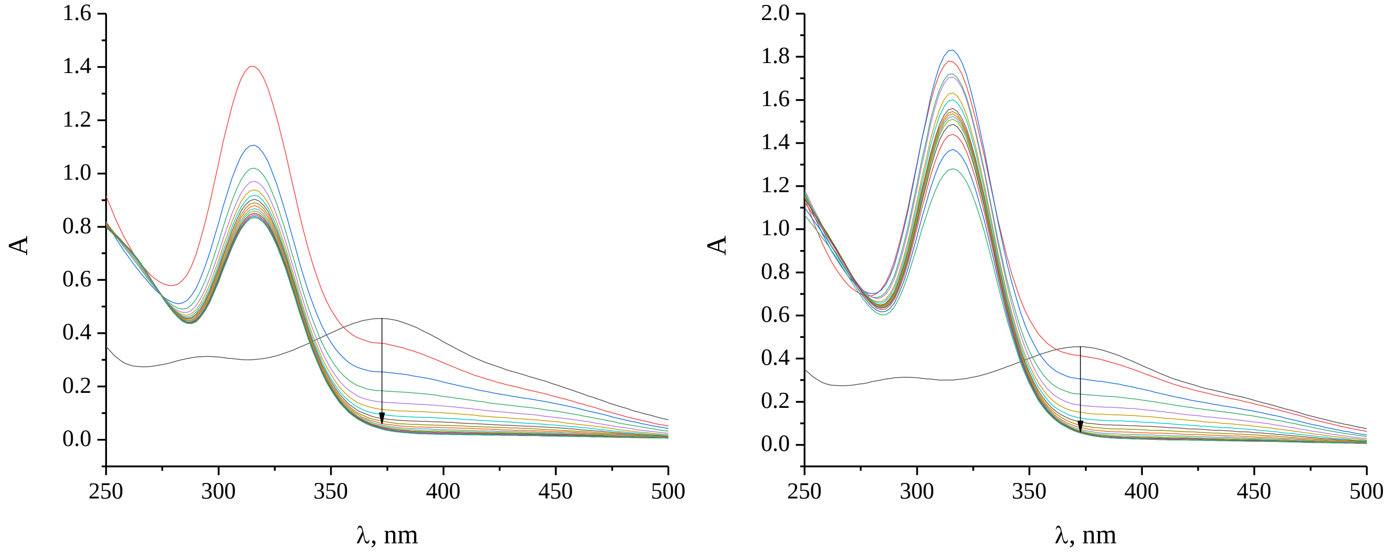

a b

Figure S8. Absorbance spectra of **L** (10 µM) before and after reduction by SDT (1.0 mM) under normoxic (20% O_2) (a) and hypoxic (less than 0.1% O_2) (b) conditions for 15 min, PBS buffer (pH 7.4) at 37 °C.

a

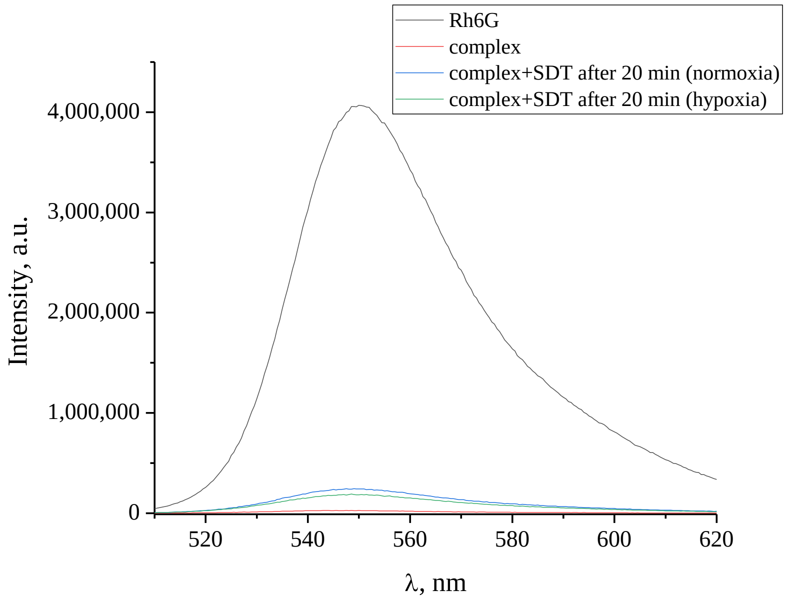

b

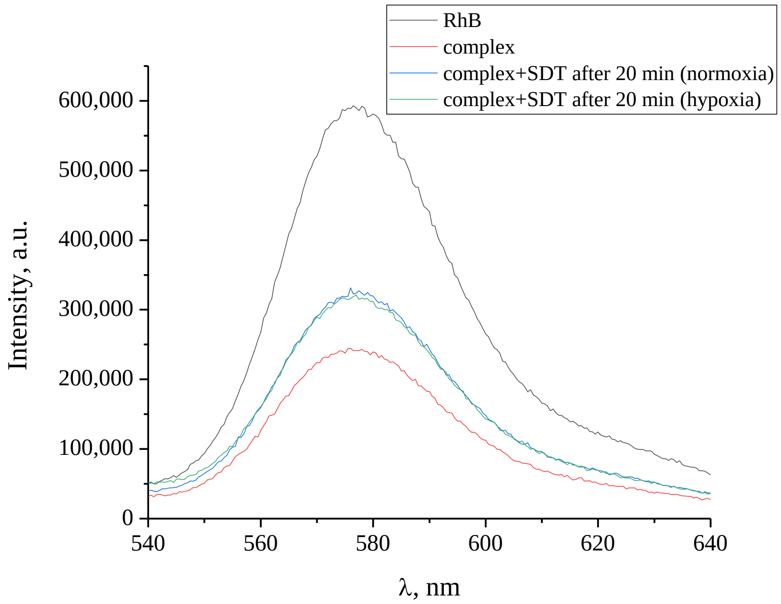

c

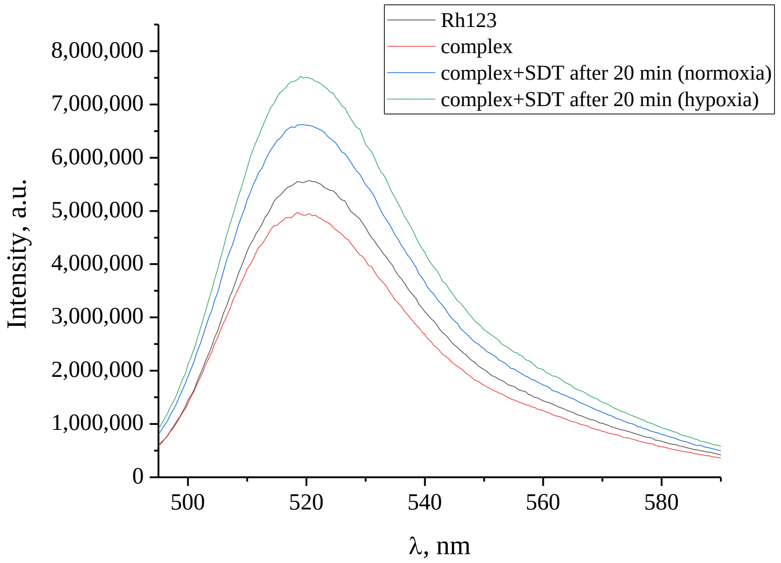

Figure S9. Fluorescent spectra of dye, the complex before the addition of SDT and after 20 minutes of exposure with the addition of SDT for systems with Rh6G (**a**), RhB (**b**) and Rh123 (**c**). C [dye] = 1 μM, C [**L**] = 1 μM, C [SDT] = 100 μM, PBS buffer (pH 7.4) at 37 °C.
